# Emerging Therapies for Advanced Clear Cell Renal Cell Carcinoma

**DOI:** 10.15586/jkcvhl.2020.156

**Published:** 2020-12-14

**Authors:** Alexander T. Toth, Daniel C. Cho

**Affiliations:** 1 Senior Clinical Research Coordinator, Phase I Drug Development Program, The Laura and Isaac Perlmutter Cancer Center at NYU Langone Medical Center, New York, NY, USA; 2 Director, Phase I Drug Development Program, The Laura and Isaac Perlmutter Cancer Center at NYU Langone Medical Center, New York, NY, USA

**Keywords:** HIF, immunotherapy, renal cell carcinoma, systemic therapy, TKI

## Abstract

Multiple combinational regimens have recently been approved and are now considered the standard of care for patients with advanced clear cell renal cell carcinoma (RCC). Several additional combinational regimens are deep in clinical assessment and are likely to soon join the crowded front-line therapeutic landscape. Most of these regimens are combinations of agents already approved as single-agents in RCC including tyrosine kinase inhibitors (TKI) and immune checkpoint inhibitors. While these new front-line regimens are associated with reliably high response rates and prolonged survival, complete and durable remissions remain limited to a small subset of patients and the vast majority of patients continue to require subsequent therapy. The need for the continued development of novel agents in RCC persists and efforts have focused on agents targeting the molecular biology of clear cell RCC and novel immunotherapies including cytokines. In this review, we discuss the progress in the development of these novel therapies in the context of the evolving standard of care for patients with advanced clear cell RCC.

## Introduction

Renal cell carcinoma (RCC) is the seventh most common malignancy in the USA with over 73,000 cases and 14,830 deaths expected in 2020 (1). The majority of patients present with localized disease, however, approximately one-third present with locally advanced or metastatic disease and up to 30% of patients undergoing surgical resection will experience recurrent disease (2). Before 2006, the only available treatments for advanced clear cell RCC were interferon (IFN)-α and interleukin-2 (IL-2), both cytokine therapies associated with serious toxicity and limited efficacy (3). Between 2006 and 2010, the approval of multiple tyrosine kinase inhibitors (TKI) contributed to a doubling in the median survival time from around 15 to 30 months (4). The following several years has seen the rapid development and approval of next-generation TKIs (cabozantinib and lenvatinib) as well as the approval of immune checkpoint inhibitors (nivolumab, ipilimumab, and pembrolizumab) either as single agents or in combinational regimens. In light of the numerous recent changes to the RCC therapeutic landscape, we will briefly review the molecular biology of clear cell RCC and the current standard of care for advanced clear cell RCC and then discuss emerging therapies in this rapidly evolving context. The primary objective of this review is to provide insight into how the novel therapies currently under investigation in clear cell RCC have evolved from our understanding of the biology of this disease as well as the clinical experience with standard agents thus far.

## Molecular Biology of Clear Cell RCC

While RCC is a heterogeneous disease, the clear cell histologic subtype remains the most common representing approximately 70% of all renal tumors and an even larger proportion of patients with metastatic RCC (>80%) (4–6). The vast majority of clear cell RCC is characterized by biallelic loss of function of the von Hippel Lindau (VHL) tumor suppressor gene. Loss of the short arm of chromosome 3, which

contains the *VHL* gene, has been found in 70–90% of clear cell RCC and is believed to be the earliest event in tumorigenesis (7, 8). The loss of function of the remaining copy of *VHL* subsequently occurs by somatic mutation, deletion, or epigenetic silencing. VHL forms a part of the oxygen-dependent VHL E3 ubiquitin ligase complex which functions to promote the ubiquitinylation and subsequent proteasomal degradation of hydroxylated proteins (9). The Cancer Genome Analysis (TCGA) for RCC also characterized low-frequency mutations in other members of the VHL complex (e.g., *TCEB1, CUL2*) in clear cell RCC not possessing alterations in *VHL*, suggesting that loss of function of the VHL complex is a central feature of nearly all clear cell RCC (10). Two of the best-studied target proteins for the VHL complex are the transcription factors—hypoxia-inducible factors, (HIF)-1α and -2α (11). Loss of VHL function results in accumulation of both HIF-1α and HIF-2α and the subsequent activation of their target genes, including vascular endothelial growth factor (VEGF), platelet-derived growth factor (PDGF), transforming growth factor-alpha (TGF-α), erythropoietin, GLUT-1, and CXCR4 (12–14).

In addition to the loss of chromosome 3p and subsequent loss of VHL, another critical event in clear cell RCC tumorigenesis is the gain of chromosome 5q, which occurs in 67% of RCC, and loss of chromosome 14q, occurring in 45% (8). Studies have also shown that loss of 3p and gain of 5q can occur simultaneously through chromothripsis in up to 28% of RCC and this may be the initial tumorigenic event in these tumors (15). Clear cell RCC is also characterized by lower frequency somatic mutations in chromatin remodeling genes, including PBRM1, BAP1, and SETD2 (present in 38.0, 11.0, and 13.2%, respectively) (8). While these mutations appear to have prognostic value (PBRM1 loss appears to be associated with favorable outcomes while BAP1 loss is associated with unfavorable outcomes), the predictive value with respect to specific therapies continues to be explored (16, 17). For example, a recent study has shown that PBRM1 loss may be associated with reduced clinical benefit in response to immunotherapy due to a decrease in STAT1 phosphorylation and subsequent enhancement of IFNγ gene expression (18).

## Recent Developments in Systemic Therapy for Clear Cell RCC

Most patients with newly diagnosed advanced clear cell RCC will immediately begin systemic therapy. However, an area of controversy in the field remains, the role of cytoreductive nephrectomy for the subset of patients presenting with metastatic RCC and primary tumor in place. Historically, the standard of care for clinically appropriate patients was to undergo a debulking nephrectomy based on the results from a randomized Phase II trial in which patients randomized to receive a nephrectomy followed by IFNα had superior survival compared with those randomized to IFNα alone (19). Recent studies in the TKI era have challenged this practice. The SURTIME trial found that patients randomized to receive sunitinib followed by delayed cytoreductive nephrectomy had improved overall survival (OS) compared with those randomized to undergo immediate cytoreductive nephrectomy followed by sunitinib, although there was no significant difference in progression-free survival (PFS) (20). As OS was a secondary endpoint, the study was felt to be underpowered to draw a definitive conclusion based on this endpoint. The CARMENA study randomized 450 patients to either sunitinib alone or cytoreductive nephrectomy followed by sunitinib and found that sunitinib alone was not inferior to cytoreductive nephrectomy followed by sunitinib with respect to OS (21). Following this, a subsequent analysis suggested that there may be a benefit for cytoreductive nephrectomy for patients with only one risk factor by International Metastatic Renal Cell Database Criteria (IMDC) (22). Thus, currently, the decision on whether or not to undergo a debulking nephrectomy remains a clinical decision and the value of this approach with frontline immunotherapy remains unclear. The Southwest Oncology Group (SWOG) has initiated a study randomizing patients who are neither immediate progressors nor complete responders following induction immunotherapy to either cytoreductive nephrectomy followed by systemic therapy or systemic therapy alone. This study will hopefully add more clarity on the value of cytoreductive nephrectomy in the context of immunotherapy.

Once patients start systemic treatment, the standard treatment for clear cell RCC for many years consisted of sequential, mostly single-agent therapy with either immunotherapy, VEGF-targeted agents, or inhibitors of mammalian target of rapamycin (mTOR). These agents are outlined in Table 1. In the last 3 years, three combinational regimens have been approved for first-line therapy which have reshaped the therapeutic landscape.

**Table 1:** Approved drugs and regimens for patients with advanced RCC.

Drug/Regimen	Mechanism of action	FDA approval date	NCCN category
**Sorafenib**	TKI targeting VEGFR, PDGFR, KIT, RAF	12/20/2005	2B: subsequent line, all risk groups
**Sunitinib**	TKI targeting VEGFR, PDGFR, KIT, RET	01/26/2006	2A: first and subsequent line, all risk groups
**Temsirolimus**	mTOR inhibitor	05/30/2007	2A: first line in poor/intermediate risk groups; 2B: subsequent line all risk groups
**Everolimus**	mTOR inhibitor	03/30/2009	2A: subsequent line all risk groups
**Bevacizumab + IFNα**	Monoclonal Ab against VEGF	07/31/2009	2B: subsequent line all risk groups
**Pazopanib**	TKI targeting VEGF, PDGFR, KIT	10/19/2009	2A: first and subsequent line, all risk groups
**Axitinib**	TKI targeting VEGFR 1-3, KIT, PDGFR	01/27/2012	1: subsequent line all risk groups; 2B: subsequent line poor/intermediate-risk groups
**Nivolumab**	Monoclonal Ab against PD-1	11/23/2015	1: subsequent line all risk groups
**Cabozantinib**	TKI targeting VEGFR2, MET, AXL, RET	04/24/2016	1: subsequent line all risk groups; 2A: first-line poor/intermediate-risk groups; 2B: first-line good risk
**Lenvatinib +/- Everolimus**	TKI targeting VEGFR 1-3, FGFR, PDGFR, KIT, RET	05/13/2016	1: subsequent line all risk groups
**Ipilimumab + Nivolumab**	Monoclonal Ab against CTLA4 and PD-1	04/16/2018	1: first-line poor/intermediate-risk; 2A: first and subsequent line all risk groups
**Axitinib + Pembrolizumab**	TKI plus PD-1 Ab	04/19/2019	1: first-line poor/intermediate-risk; 2A: first and subsequent line all risk groups
**Axitinib + Avelumab**	TKI plus PD-L1 Ab	05/14/2019	2A: first-line all risk groups; 3: subsequent line all risk groups

### Ipilimumab plus nivolumab

The combination of nivolumab (a monoclonal antibody (Ab) targeting programmed death (PD)-1) and ipilimumab (a monoclonal Ab targeting cytotoxic T-lymphocyte-associated protein (CTLA-4)) was assessed in a randomized Phase III trial versus sunitinib in patients with untreated advanced RCC (23). The initial publication reported the results of 847 patients with intermediate or poor-risk features by IMDC risk category. Patients who had received the combination of ipilimumab and nivolumab experienced superior objective response rate (ORR) (42 vs 27%; P < 0.001) and 18-month overall survival (OS) rate (75 vs 60%; 95% CI 55 to 65) compared with those treated with sunitinib. At the time of the most recent update after 42 months of follow-up, patients with intermediate or poor-risk RCC treated with ipilimumab and nivolumab had experienced a superior median OS (47.0 vs 26.6 months; HR 0.66; 95% CI 0.55 to 0.80; P < 0.0001), PFS (11.6 vs 8.3 months; HR 0.75; 95% CI 0.62 to 0.90; P < 0.0015), and maintained superior ORR (42.1 vs 26.3%; P < 0.001) (24). In patients with favorable risk, similar advantages to ipilimumab and nivolumab were not observed with patients treated with the combination experiencing ORR of 28.8 versus 54.0% (P < 0.0001) and HR for death of 1.19 (95% CI 0.77 to 1.85) compared with patients treated with sunitinib. Across all risk categories, patients treated with ipilimumab and nivolumab had a greater median duration of response (NR vs 24.8 months; HR 0.48; 95% CI 0.34 to 0.67; P < 0.0001) and more complete responses (10.1–12.8% vs 1.4–5.6%, intermediate/poor risk–favorable risk) compared with sunitinib. Based on the initial results, the United States Food and Drug Administration (FDA) approved the combination of ipilimumab and nivolumab as first-line treatment for patients with intermediate/poor-risk advanced RCC in April 2018 and this treatment now carries a category 1 recommendation for intermediate/poor-risk RCC and category 2A recommendation for favorable risk RCC as per the guidelines of the National Comprehensive Cancer Network (NCCN) (19, 25).

### Axitinib plus pembrolizumab

The combination of axitinib and pembrolizumab (a monoclonal Ab targeting PD-1) was assessed in a randomized Phase III trial versus sunitinib in patients with advanced RCC (KEYNOTE-426) (26). Overall, 861 patients were randomized to receive either the combination of axitinib and pembrolizumab or sunitinib. Patients who received the combination of axitinib and pembrolizumab experienced significantly better median PFS (15.1 vs 11.1 months; HR 0.69; 95% CI 0.38 to 0.74; P < 0.001), ORR (59.3 vs 35.7%; P < 0.001), and 12-month survival rate (89.9 vs 78.3%, HR 0.53; 95% CI 0.38 to 0.74; P < 0.0001). The benefit of axitinib and pembrolizumab over sunitinib was observed across all IMDC risk groups and was irrespective of PD-L1 expression. Based on these results, the combination of axitinib and pembrolizumab was approved by the FDA in April 2019 for patients with advanced RCC regardless of IMDC risk group and currently is given a category 1 recommendation by the NCCN for first-line therapy.

### Axitinib plus avelumab

The combination of axitinib and avelumab (monoclonal Ab targeting PD-L1) was similarly assessed in a large randomized Phase III trial versus sunitinib in patients with advanced RCC in which 886 treatment-naïve patients were randomized to receive either the combination or sunitinib (JAVELIN). The study was designed with the primary endpoints of PFS and OS among patients with PD-1 Ligand (PD-L1) positive tumors. In the initial report of the results, the combination of axitinib and avelumab resulted in superior PFS in both patients with PD-L1 positive tumors (13.8 vs 7.2 months; HR 0.61, 95% CI 0.47 to 0.79; P < 0.001) and the overall population (13.8 vs 9.4 months; HR 0.69; 95% CI 0.56 to 0.84; P < 0.001) compared with sunitinib (27). The combination also resulted in superior ORR in both the PD-L1 positive population (55.2 vs 25.5%) and in the overall study population (51.4 vs 25.7%). The clinical benefits were observed across all IMDC risk groups. At the time of the most updated results, OS data were still not mature (28). Based on these results, the combination of axitinib and avelumab was approved by the FDA in May 2019 for patients with advanced RCC regardless of IMDC risk group and is also currently given a category 1 recommendation by the NCCN for first-line therapy.

## Subsequent Therapy

The most current standard care for the majority of patients with advanced clear cell RCC will be to start treatment with combinations of ipilimumab–nivolumab, axitinib–pembrolizumab, or axitinib–avelumab. However, as noted above, complete responses that are maintained off therapy are rare and the vast majority of patients will require subsequent lines of therapy. With the changes to frontline therapy being so recent, the most appropriate choice of second-line therapy remains unclear. A small retrospective analysis has shown that the combination of ipilimumab–nivolumab has moderate activity in patients treated with prior therapy including the combination of TKI–checkpoint inhibitors (29). As patients treated initially with ipilimumab–nivolumab are TKI-naïve, logical second-line options would include single-agent TKI and TKI–checkpoint inhibitor combinations. This remains an area that needs novel therapeutic development and we will review some progress in this regard in the following sections.

Determination of the most appropriate sequence of the agents will require continued efforts to develop predictive biomarkers of response to current therapies. While significant effort has gone into identifying mechanisms of resistance to VEGF-targeted therapies, whether these same pathways will be relevant when VEGF-targeted therapies are combined with immune therapy remains unclear (30). Similarly, no clear predictors of resistance to the combination of ipilimumab and nivolumab have been identified.

## First-line Trials in Progress with TKI plus Immunotherapy

Several important clinical trials are underway which may also contribute to the first-line RCC treatment paradigm (Table 2). Many of these trials are assessing combinations of second-generation TKI with PD-1 antibodies to build on the positive results from the KEYNOTE-426 and JAVELIN studies. Positive results were recently announced for the CheckMate-9ER which is a randomized Phase III trial of cabozantinib plus nivolumab versus sunitinib in patients with previously untreated advanced clear cell RCC (31). In a press release, it was announced that patients treated with the combination of cabozantinib and nivolumab experienced a superior PFS, OS, and ORR compared with patients treated with sunitinib. The CLEAR trial is a randomized Phase III of the combination of lenvatinib with everolimus or pembrolizumab versus sunitinib in previously untreated patients with advanced clear cell RCC that has completed accrual. Finally, the COSMIC-313 trial is a randomized Phase III trial of cabozantinib in combination with iplimumab and nivolumab versus ipilimumab and nivolumab in previously untreated patients with advanced clear cell RCC with poor or intermediate risk factors. Positive results from any of these studies would be expected to result in approvals of new combinational regimens in the first-line setting. In the current paradigm, practitioners are choosing between a pure immunotherapy regimen (ipilimumab/nivolumab) versus the combination of a TKI and PD-1/PD-L1 Ab. However, compelling results from the combination of cabozantinib with ipilimumab and nivolumab in the COSMIC 313 trial might establish this combination as the dominant option in first-line treatment.

**Table 2:** Important ongoing clinical trials in patients with advanced RCC.

Title	Drugs/Design	Line of therapy
Checkmate-9ER	Randomized Phase III trial of cabozatinib plus nivolumab versus sunitinib	First
CLEAR	Randomized Phase III trial of lenvatinib with everolimus or pembrolizumab versus sunitinib	First
COSMIC-313	Randomized Phase III trial of cabozantinib plus ipilimumab and nivolumab versus ipilimumab and nivolumab	First
MK-6482-005	Randomized Phase III trial of MK6482 versus everolimus	Second–Fourth
CANTATA	Randomized double-blind Phase II trial of CB-839 plus cabozatinib versus cabozanitinib	Second–Third
PIVOT-09	Randomized Phase III trial of bempegaldesleukin plus nivolumab versus sunitinib or cabozantinib	First

## Novel Agents in Development in RCC

While the studies discussed thus far involve combinational regimens involving agents that are already approved in RCC, there are also numerous agents in clinical development in clear cell RCC with completely novel mechanisms of action. Like the VEGF-targeted therapies, many of these novel agents target therapeutic vulnerabilities engendered by the molecular biology of clear cell RCC.

### HIF2 inhibitors

The novel agents perhaps most centrally targeted to the molecular biology of clear cell RCC are inhibitors of HIF-2α. Numerous studies have established that HIF-2 is the more relevant HIF isoform in terms of RCC tumorigenesis and progression (32–35). While numerous strategies have been developed to target the HIFs, one which has emerged are allosteric inhibitors which bind to the PAS domains in HIF-2α preventing its association with HIF-2β and subsequent activation. PT2385 was the first such inhibitor of HIF-2α to enter clinical assessment. In a recently reported Phase I trial, 51 heavily pretreated patients with advanced clear cell RCC were enrolled to either the dose-escalation (26 patients) or expansion phase (25 patients) (36). PTC2385 was well tolerated and ORR for the entire group was 14%. Clinical development continued with the closely related PT2977 (MK6482) which, following a Phase I dose-escalation trial establishing the recommended Phase II dose, was recently assessed in an open-label Phase II trial in patients with advanced clear cell RCC with at least one prior treatment (37, 38). Overall, 55 patients were enrolled and treated at a dose of 120 mg PO daily. The ORR was reported to be 24% with a disease control rate, (Patients with stable disease + patients with partial responses + patients with complete responses) of 80%, and median PFS was 11 months. The activity was observed across all IMDC risk groups. The results of a Phase II trial of MK6482 in patients with VHL syndrome and renal tumors were recently reported as well (39). Overall, 61 patients were enrolled in the study and treated with MK6482, also at 120 mg PO daily. At the time of the report, there were 27 (27.89%) confirmed responses and eight (13.1%) unconfirmed responses. The median duration of response and PFS had not yet been reached. Based on these results, MK6482 was granted breakthrough designation for patients with VHL disease-associated RCC in June 2020. MK6482 is currently being assessed in multiple Phase III trials as well as multiple combinations.

Another strategy to target HIF-2α is to reduce expression through RNA interference (RNAi). The development of RNAi-based therapies has been challenged by delivery, poor bioavailability due to rapid degradation, poor cellular penetration, and preferential absorption by liver tissue. Arrowhead Therapeutics has developed a next-generation RNAi-based therapy directed against HIF-2α (ARO-HIF2) using a polyconjugate system designed to deliver to the tissue outside the liver. ARO-HIF2 is currently being assessed in a Phase Ib trial in patients with advanced clear cell RCC (NCT04169711).

### Glutamine antagonists

Similar to many other malignancies, clear cell RCC has been characterized by alternations in numerous metabolic pathways (40). In particular, clear cell RCC is believed to depend on the availability of glutamine due to a dependency on reductive carboxylation, a process in which glutamine is metabolized to form citrate for lipid biosynthesis (41). Glutamine must be converted to glutamate in order to be utilized by the cell. Many have proposed the hypothesis that the so-called “glutamine addiction” observed in clear cell RCC may be a therapeutic vulnerability that might be exploited through treatment with glutaminase inhibitors. Telaglenastat (CB-839) is a selective oral inhibitor of glutaminase which has shown preliminary activity in patients with RCC in combination with both cabozantinib and everolimus. In the initial Phase I trial of the combination, 27 pretreated patients with both clear cell and papillary histology were enrolled and treated with the combination of CB-839 and everolimus. Of 24 evaluable patients at the time of report, one patient experienced a PR while 21 had the best response of SD for an overall disease control rate of 92% (42). For the combination of CB-839 and cabozantinib, 13 heavily pretreated patients were enrolled and treated with the combination. Among 12 evaluable patients, five patients experienced a PR (ORR 42%) and seven had the best response of SD for a disease control rate of 100% (43). A randomized placebo-controlled Phase II trial (CANTATA) of CB-839 plus cabozantinib versus cabozantinib has been initiated and completed accrual although no results have yet been reported (NCT03428217). Based on the Phase I trial results, the combination of CB-839 and cabozantinib was granted Fast Track designation by the FDA in May 2018.

### Adenosine 2A receptor inhibitor

Much effort has been directed towards enhancing the efficacy of immune checkpoint inhibitors in RCC. Preclinical studies have shown that adenosine accumulation in the tumor microenvironment leads to the suppression of the immune activity of cytotoxic T-cell and natural killer (NK) cells and enhancement of the activity of regulatory T-cells (Tregs) and myeloid-derived suppressor cells (MDSC) (44–46). Adenosine modulates the activity of immune cells by binding to adenosine 2A receptor (A2AR) on the cell surface and has been implicated as a potential resistance mechanism to checkpoint inhibitors (47). Ciforadenant (CPI-444) is a small molecule that binds to A2AR and competitively inhibits its interaction with adenosine. Ciforadenant was recently assessed in a Phase I trial alone and in combination with atezolizumab (PD-L1 Ab) (48). Overall, 68 patients with pretreated advanced RCC were enrolled and treated with either ciforadenant monotherapy (33 patients) or the combination (35 patients). Most (72%) patients had previously been shown to be resistant or refractory to prior PD-1/PD-L1 Ab therapy. Four of 35 patients treated with the combination experienced PR (ORR 11%). Median PFS for the combination was 5.8 months. Appended correlative studies suggested that the efficacy of ciforadenant was associated with enhancement of immune function as noted by increased CD8+ T-cell infiltration and diversification of the TCR repertoire. The overall activity of the combination, in particular, was felt to be promising particularly in this heavily pretreated and largely checkpoint inhibitor-resistant/refractory, PD-L1-low population. Ciforadenant is currently being developed broadly in other tumor types along with an adenosine gene signature biomarker (AdenoSig) developed as part of the original Phase I trial.

### Cytokines

The long-established efficacy of high dose (HD) interleukin 2 (IL-2) in a subset of patients with advanced RCC has continued to stoke interest in the potential of cytokine therapy in this disease. Although HD IL-2 has been shown to reliably induce durable and complete remissions in a small group of patients with RCC, this therapy has been limited by its severe toxicity requiring inpatient delivery in specialized centers. Efforts continue to identify predictive models to limit its application to those most likely to benefit. In parallel, pharmaceutical companies have focused on developing novel agents that can both enhance the efficacy of IL-2 and reduce toxicity to allow outpatient administration. Bempegaldesleukin (NKTR-214) is a pegylated CD122 preferential IL-2 agonist designed to stimulate immune cells through the IL2βγ receptor with a limited engagement of the IL2αR subunit to stimulate CD8+ T and NK cells without the accompanying proliferation of Tregs observed with unmodified IL-2. After completing Phase I single-agent assessment, bempegaldesleukin was recently studied in combination with nivolumab in patients with select solid tumors including RCC (49, 50). Of 14 patients with previously untreated clear cell RCC treated with the combination in either the dose escalation or expansion cohorts, 10 experienced a partial or complete response for an ORR of 71.4%. Based on these results, this combination is currently being assessed in a randomized Phase III trial compared with sunitinib or cabozatinib in patients with untreated advanced clear cell RCC (NCT03729245). Based on the results observed in patients with melanoma, bempegaldesleukin was granted Breakthrough status by the FDA in August 2019 and its clinical development is moving forward in numerous cancers including RCC.

There are other versions of IL-2 in clinical development as well. ALKS 4230 is a recombinant formulation of IL-2 with intermediate affinity to IL2R, sparing interaction with and simulation of Tregs which are characterized by expression of high-affinity IL2R. ALKS 4230 is currently in clinical assessment in a Phase I trial as a single agent and in combination with pembrolizumab (NCT02799095) (51). The trial is ongoing and includes a cohort of patients with clear cell RCC.

In addition to IL-2, there has been increased interest in other cytokines with similar biologic activity. IL-15 is an immunostimulatory cytokine whose receptor shares the same β and γ chains as the IL-2 receptor but contains a distinct α chain whose engagement results in stimulation of T cells and NK cells and maintenance of memory CD8+ T cells without expansion of T-regs (52). The development of unmodified IL-15 has been limited by toxicity and bioavailability and most strategies have focused on modified versions. AL-803 is a complex containing two IL-15 agonist domains associated with two IL-15α receptor domains fused to a human IgG1 Fc which prolongs the half-life of the complex. AL-803 has recently been assessed in a Phase I trial including patients with advanced RCC in both subcutaneous and intravenous administrations (53). While no significant objective responses were observed, ALT-803 was associated with only mild cytokine-related toxicities and there was evidence of expected T-cell and NK-cell expansion. ALT-803 then showed promising efficacy in a small Phase I trial in combination with nivolumab in patients with NSCLC (54). Further development in other tumor types including RCC continues.

IL-12 is another proinflammatory cytokine that stimulates NK and T-cells to enhance proliferation and IFN-γ production as well as to promote differentiation of T-cells into T helper 1 (Th1) cells (55). While the utilization of recombinant IL-12 as a therapy for advanced cancers has had a strong rationale, its application has been limited by toxicity, bioavailability, and a narrow therapeutic window induced by an apparent adaptive response to attenuate IL-12 effects (56). NHS-IL12 is an engineered immunocytokine in which two IL-12 heterodimers have been fused to the NSH76 antibody which targets regions of tumor necrosis to enhance on-target exposure and limit systemic toxicity. NHS-IL12 was recently assessed in a Phase I trial in patients with advanced malignancies (57). While no objective tumor responses were observed, pharmacodynamics studies showed evidence of increased T-cell receptor diversity and tumor-infiltrating lymphocytes (TIL) density. NHS-IL12 is currently being assessed in combination with avelumab in patients with solid tumors including patients with advanced RCC.

### Novel checkpoint inhibitors

Building upon the efficacy of the antibodies against PD-1 and CTLA4, a bewildering array of other agents directed against other immune checkpoints have been developed and entered clinical assessment. These include agonistic antibodies against co-stimulatory checkpoint pathways (e.g., CD27, OX40, ICOS, 4IBB) and blocking antibodies against co-inhibitory checkpoint pathways (e.g., LAG3, TIM3/Galectin-9, TIGIT, GITR, Siglec15, VISTA, HLA-G/ILT4, BTLA). While none of these novel checkpoint inhibitors have entered dedicated RCC clinical trials, some preliminary activity in early phase clinical trials has been observed. For example, utomilumab, an agonistic antibody against 4IBB recently demonstrated promising activity in combination with pembrolizumab (58). Out of five patients treated with RCC, one patient experienced a CR while another experienced a PR Translational studies have suggested that PD-1 and LAG3 are the most frequently overexpressed checkpoints in combination on circulating T-cells of patients with RCC (59). While a combination of BMS-986016 (LAG3 Ab) with nivolumab has shown promising activity in melanoma, similar results have not yet been disclosed in RCC (60). In general, the vast majority of these novel checkpoints are continuing in clinical development in combination with PD-1/PD-L1 antibodies. Promising activity seen in early phase trials and dedicated expansion cohorts will hopefully soon inform Phase II and III trials in RCC.

## Future Perspective

The therapeutic bar in advanced clear cell RCC has been raised high in the first-line setting by the recent and expected approvals of multiple combinational regimens. In general, these regimens can reliably produce high response rates and prolonged responses. Nonetheless, complete responses that are durable off-therapy remain rare. Progress in developing new first-line regimens must therefore focus on enhancing the quality of response in terms of depth and durability rather than on incremental increases in response rate or PFS. Towards this end, a greater focus is expected on novel immunotherapies, particularly cytokines. With the vast majority of patients still requiring sequential therapies, there remain significant opportunities to assess novel agents. The novel inhibitors of HIF-2α present the most exciting opportunity to target the central molecular biology of RCC in rational combinations with other agents.

## Conclusions

Significant improvements have been made in the therapy of patients with advanced clear cell RCC and recently approved combinational regimens have changed the standard of care in the first-line setting. Ongoing Phase III trials will very likely add new combinational regimens to the standard options currently available. Despite this progress, the paucity of complete and durable remissions resulting from treatment with current standard regimens highlights the need to continue to develop novel agents. As we have outlined, the development of novel inhibitors of HIF-2α and glutaminase continues to provide hope that agents targeting the fundamental biopsy of clear cell RCC will improve patient outcomes. In parallel, a multitude of novel immunotherapies seeks to build on the established efficacy of cytokines and checkpoint inhibitors in this disease. While prolonged responses on sequential therapies are in clear progress, the goal remains to cure more patients with advanced clear cell RCC and achievement of this goal requires a commitment to novel therapeutic development.
